# Institutional Review of Hemorrhagic Pelvic Emergencies Effectively Managed with Percutaneous Arterial Embolization

**DOI:** 10.7759/cureus.2194

**Published:** 2018-02-15

**Authors:** Muhammad Azeemuddin, Raza Sayani, Nauman Turab, Syed M Mustahsan, Mohammad Hasan, Dawar B Khan, Fatima Mubarak

**Affiliations:** 1 Department of Radiology, The Aga Khan University, Karachi.; 2 Radiology Department, Dow University of Health Sciences (DUHS), Karachi, Pakistan; 3 Emergency Medicine, The Aga Khan University Hospital, Karachi.; 4 Medical Student, Jinnah Sindh Medical University (SMC); 5 Department of Radiology, The Aga Khan University, Karachi

**Keywords:** angioembolization, pelvic injuries, hemorrhage, internal iliac artery

## Abstract

Objective: Our aim was to review the results of pelvic arterial embolization (PAE) performed in the interventional radiology suite.

Method: The data of all patients in whom pelvic angioembolization was performed was collected from July 2011 to June 2017. Procedures were performed by an experienced interventional radiologist. The clinical and laboratory data, as well as the outcome data, were obtained from the medical records of our hospital. The following parameters were collected for each patient, including the age, gender, presenting symptoms, site of bleeding, catheters used for embolization, material used for embolization, previous computed tomography (CT) scan and/or focused assessment with sonography for trauma (FAST) ultrasound, average hemoglobin before the procedure, and patient clinical status on discharge.

Result: A total of 37 patients underwent pelvic angiography for acute hemorrhage at our institution. They had contrast blush, active extravasation, or abnormal vascularity from the branches of the internal iliac artery and underwent therapeutic transcatheter embolization. A total of 29 patients (78.3%) were male and 8 (21.7%) were female. The average age was 30.0 years (range: 6-90 year). Of these, 16 patients (43.2%) presented with road traffic accidents (RTAs), six with gunshot injuries (16.2%), six with iatrogenic injuries (16.2%), four with a history of a fall (10.8%), two with bomb blast injuries (5.4%), one with a history of a glass injury (2.7%), one had a history of a roof falling on her during an earthquake, and one patient had a pelvic pseudoaneurysm secondary to an abscess. The type of embolic material used for embolization included coils in 16 patients, polyvinyl alcohol* *(PVA) particles were used in eight patients, both PVA particles and coils were used in 11 patients, and glue was used in one patient. All were successfully embolized. Thirty-four were discharged while three patients expired during the course of hospital stay due to other coexisting morbidities.

Conclusion: The management of pelvic injuries has always been a topic of debate, with multiple methods reported to date but growing evidence supports the use of pelvic arterial embolization in hemorrhagic pelvic injuries. The formulation of a standardized protocol is the need of the day.

## Introduction

Pelvic fractures are common, difficult to manage, and carry a high risk of disability or death [1]. Most pelvic fractures in our population are due to motorcycle accidents. The patients are often young males. These fractures are usually not in isolation but are associated with multiple injuries to the chest and abdomen due to the high nature of the impact, making the situation more complex. They are often associated with vascular injuries. The management of arterial bleeding in the pelvis remains a challenge for emergency trauma care and carries high morbidity as well as mortality [2]. Surgeons may be able to fix multiple and difficult fractures with expertise; however, a small bleeding vessel in the pelvis may not only be difficult to localize due to the physical constraints of the anatomical space but can also lead to hemodynamic instability.

Arterial embolization (AE) is a less invasive procedure that supplements operative management for the treatment of pelvic hemorrhages [3]. The technique was described in 1972 by Margolies and colleagues and is reported to be effective in patients bleeding from small arterial branches of the internal iliac artery. Increasingly, AE is used as a nonoperative method to address injury after blunt trauma in hemodynamically stable patients [4], whereas pelvic packing is an effective intervention in hemodynamically unstable patients. AE is also helpful in post-surgical cases where major surgeries, such as tumor resection etc., have been successfully performed; however, small vascular bleeds may result as a complication. Despite its widespread use, complications of the technique are rarely reported [5]. This retrospective review aims to share our results and create awareness of pelvic angioembolization in the local community as an effective modality for controlling arterial hemorrhage in pelvic injuries.

## Materials and methods

A retrospective cohort study was conducted in the Radiology department for a period of six years from July 2011 to June 2017. The data was extracted from the electronic medical record system. Study variables included demographics, data on the type and cause of injury, active extravasation on a previous computed tomography (CT) scan, initial hemoglobin before the procedure, site of bleeding, catheters used for embolization, type of embolic agents used for embolization, complications of angiography and embolization, and outcome.

All patients who had undergone pelvic angiography and embolization due to active bleeding secondary to pelvic pathologies irrespective of cause, be it trauma or iatrogenic, were included. Patients who were coded as do not resuscitate (DNR), comfort care, or those who had not given consent were excluded. The patients mostly presented with trauma secondary to road traffic accident (RTA), bomb blast, gunshot, or a history of falls. Some cases that were iatrogenic with bleeding secondary to surgery or any other intervention that resulted in significant bleeding were also included.

**Figure 1:**
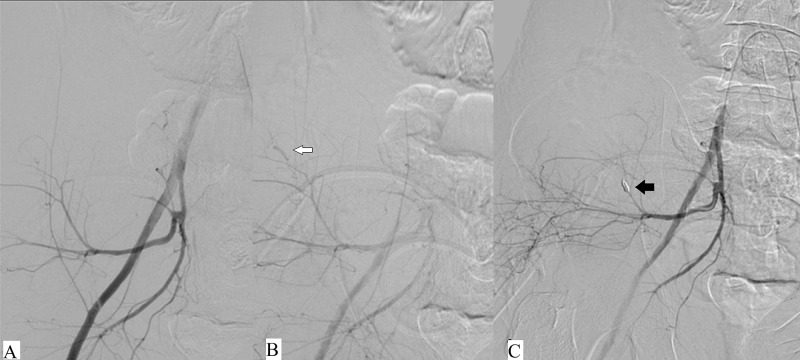
A and B: Pre-embolization runs show the right common iliac artery angiogram with active extravasation (white arrow) from the posterior division of the internal iliac artery. C: Successful embolization was performed using a coil (black arrow) with occlusion of the vessel.

The procedures were performed by experienced interventional radiologists and carried out in the dedicated Interventional radiology suite on a flat panel, monoplane, digital subtraction angiography machine (Axiom-Artis, Siemens, Munich, Germany). For super-selective catheterization and embolization, a micro-catheter (Progreat, Terumo, Shibuya, Tokyo, Japan) was used, which was coaxially placed as near as possible to the site of bleeding or extravasation, and the embolizing agent was injected or deployed. Embolization was performed when free extravasation, abnormal blush, or vascularity was seen. Vascular occlusion coils and PVA particles were used alone or in combination, as required, depending on the size of the vessel and accessibility. The feeder vessel was preferably occluded close to the site of extravasation, as shown in Figure 1.

**Figure 2:**
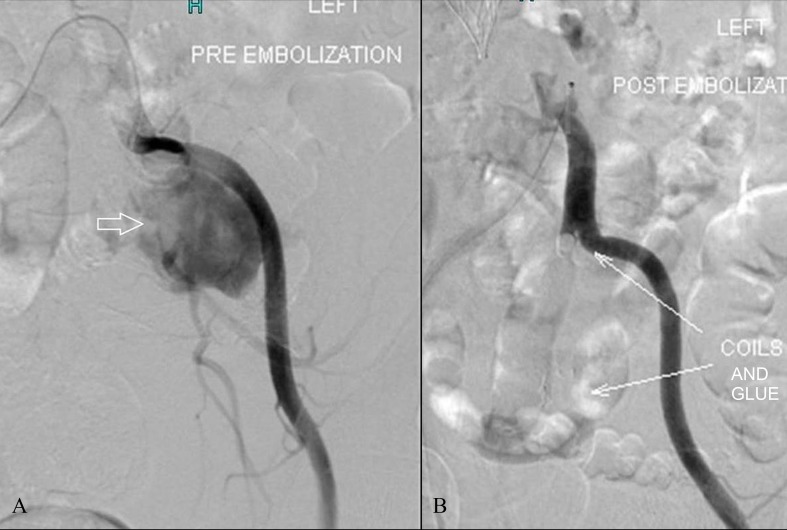
Pre- (A) and post-embolization (B) runs show the left common iliac artery angiogram showing a large pseudoaneurysm close to its origin. Successful embolization was performed using coils and histoacryl glue (arrows) with occlusion of the pseudoaneurysm.

In a few cases, embolization was performed using coils and glue, as shown in Figure 2.

## Results

A total of 37 patients underwent pelvic angiography for acute hemorrhage at our institution. All had contrast extravasation or abnormal vascularity in the pelvic vasculature and underwent therapeutic transcatheter embolization. There were 29 male (78.3%) and eight female (21.7%) patients with a male-to-female ratio of about 4:1. The average age was 30.0 years (range: 6–90 years). Sixteen patients (43.2%) presented with RTA, six with gunshot injuries (16.2%), six with iatrogenic injuries (16.2%), four with a history of falls (10.8%), two with bomb blast injuries (5.4%), one with a glass injury (2.7%), one had a history of a roof falling on her during an earthquake, and one patient had a pelvic pseudoaneurysm secondary to an abscess. In 36 patients, an abnormal blush or extravasation corresponding to the bleeding site was identified and embolized successfully. In one patient, one of the small lumbar vessels could not be cannulated; however, a major bleeder in the pelvis was successfully embolized. In one elderly female patient, embolization of the bleeding inferior epigastric artery was successfully performed. However, the vascular access site i.e. common femoral artery occluded secondary to the placement of the closure device. The stent was placed using contralateral access, resulting in the restoration of flow. The type of embolic material used for embolization included coils, which were used in 16 patients (44.4%), PVA particles were used in 8 patients (22.2%), both PVA particle and coils (30.5%) were used in 11 patients, and glue was used in one patient. Three patients expired due to other coexisting morbidities.

## Discussion

Pelvic injuries are the cause of high mortality [6]. The management of these has always been a challenging task for health care workers. External fixators, C-clamps, and belts help control hemorrhages, mainly venous; however, arterial bleeds are still difficult to control. Finally, in 1972, angioembolization was brought into practice to control pelvic bleeding, which proved to be lifesaving and efficient [7].

Pelvic arterial injuries are usually due to high-impact trauma, which, in our cases, were mainly road traffic accidents with the involvement of trucks and motorbikes in many cases. The patients were mostly young males. These injuries are usually not in isolation, involve chest and abdominal viscera, and often lead to hemodynamic instability and death. Our study showed that pelvic angioembolization has a 94.4% success rate in pelvic injuries irrespective of etiology. Along with its effectiveness, one study states some complications of pelvic angioembolization (PAE) as well, which include late bleeding, ischemia of gluteal muscles, and hemorrhage at the site of puncture [8]. No such complications were seen in the patients included in our study. One patient in whom the common femoral artery was occluded due to closure device malfunction had to be stented for flow restoration; however, he did not suffer any long-term complication.

An alternative method to prevent pelvic bleeding is pelvic packing, which is also practiced widely. It was believed that pelvic packing requires less blood transfusion than angioembolization. But, this was proved wrong by one study that showed both of the above methods require almost the same amount of blood transfusion [9].

Studies have shown that angiography can detect active extravasation and is very good for early treatment; however, it may not be able to detect the traumatized vessels. Considering this, it is important that in acute conditions, the patient should undergo surgery, but if the patient has stable hemodynamic status, we can continue with angioembolization [10]. The results of our study are convincing enough to start practicing pelvic angioembolization (PAE) for pelvic bleeding because its effectiveness cannot be denied.

A major limitation in a low-income country like Pakistan is cost. Another important drawback is the lack of trained staff and the availability of equipment, which cannot be fulfilled by every institution. Considering the advantages and effectiveness of this procedure in saving the lives of patients, we recommend that it be utilized to its maximum.

## Conclusions

Pelvic angioembolization is a growing modality in securing homeostasis in a timely manner, especially in pelvic trauma patients where bleeders are difficult to control. It can also be used alone or as an adjuvant to surgery for complex pelvic injuries or in polytrauma patients where surgery is involved. It has shown promising results but a standardized protocol for this technique is yet to be defined.
